# Synergistic interplay between triazine and curcumin or resveratrol in mitigating rotenone-induced toxicity in *Drosophila* model

**DOI:** 10.1038/s41531-026-01592-5

**Published:** 2026-11-11

**Authors:** Mai Fathy, Amal Seif, Samar El Kholy

**Affiliations:** Zoology Department, Faculty of Science, Tanta University, Tanta, Egypt

**Keywords:** Biochemistry, Drug discovery, Neurology, Neuroscience

## Abstract

Parkinson’s disease (PD) is a progressive neurodegenerative disorder characterized by dopaminergic neuronal loss, oxidative stress, and debilitating motor dysfunction. Targeting the Wnt signaling pathway provides a powerful disease-modifying strategy. Building on recent evidence that triazine derivatives modulate Wnt signaling, we used a *Drosophila* model and in silico molecular docking to validate the specific interactions driving this pathway modulation, followed by qPCR of key Wnt signaling-related genes: *armadillo*, *wingless*, and *frizzled 3*. The neuroprotective efficacy of the triazine derivative, alone or in combination with curcumin or resveratrol, was subsequently evaluated using behavioral, neurochemical, biochemical, apoptotic, immunohistochemical, and ultrastructural assessments. Treatment with the triazine derivative produced marked neuroprotective effects, which were further enhanced when combined with curcumin or resveratrol. This treatment significantly restored dopamine levels and locomotor performance, enhanced superoxide dismutase and catalase activities, and reduced lipid peroxidation compared with untreated rotenone-exposed flies. Moreover, immunohistochemical analysis indicated attenuation of apoptotic signaling, while transmission electron microscopy revealed substantial preservation of neural ultrastructure. Collectively, these findings demonstrate the neuroprotective potential of the triazine derivative and suggest that its combination with curcumin and resveratrol may provide an enhanced strategy for counteracting oxidative stress, neural apoptosis, and motor dysfunction associated with PD. The findings further support Wnt signaling modulation as a promising mechanistic target and provide a rationale for further investigations of triazine-based combination strategies as potential disease-modifying approaches for PD.

## Introduction

Neurodegenerative diseases such as Parkinson’s disease (PD) represent one of the global health challenges, particularly among the elderly. These disorders are characterized by the progressive loss of neurons, leading to severe cognitive and motor impairments that consequently reduce quality of life and increase healthcare demands worldwide. Although there is no effective cure, antioxidants such as flavonoids, polyphenols, and vitamin E and vitamin C obtained from fruits and vegetables, or nutritional supplements can help as an adjunct to pharmacological medications to prevent or ameliorate PD symptoms by fighting free radicals and reducing the effects of oxidative damage^1^ via different neuroprotective pathways.

Canonical Wnt signaling is one of these neuroprotective pathways that is critically involved in the maintenance and repair of nigrostriatal dopaminergic neurons, whose degeneration and the consequent decline in striatal dopamine (DA) levels underlie the motor and non-motor symptoms of PD^2^; boosting this pathway with small synthetic molecules represents a promising neurorestorative strategy^3,4^. Synthetic chemicals such as triazine and its derivatives have shown strong potential in modulating neuroprotective signaling pathways. Triazines can inhibit or alter GSK-3 activity, stabilize β-catenin, and promote its nuclear translocation, thereby enhancing Wnt/β-catenin–driven transcription programs that support neuronal survival and regeneration. Preclinical findings further support this potential. Sinha et al.^5^ demonstrated the neuroprotective effects of novel triazine derivatives in rodent models of Alzheimer’s disease, while studies in cancer cells revealed altered β-catenin levels following triazine treatment, reinforcing their Wnt-modulating capacity^6^. Although the triazine’s effectiveness has been established, it has not yet been evaluated in PD models.

The fruit fly *Drosophila melanogaster* is a well-established and highly informative model for investigating neurodegenerative disorders, including PD. In addition to behavioral and biochemical endpoints, the highly organized architecture of the *Drosophila* compound eye, composed of regularly arranged ommatidia containing photoreceptor neurons, rhabdomeres, and pigment cells, provides a sensitive morphological system for detecting subtle neural and cellular alterations^7–9^. Mitochondrial morphology is of particular interest because rotenone, a mitochondrial complex I inhibitor widely used to induce PD-like phenotypes, promotes mitochondrial dysfunction and oxidative stress, which may be reflected by alterations in mitochondrial size, shape, membrane integrity, and cristae organization^10,11^. Ultrastructural evaluation of the compound eyes and brain can therefore provide evidence of neurodegenerative damage.

Previous studies have successfully identified and validated antioxidant candidates in both sporadic and familial PD models using *Drosophila* (reviewed in Fathy et al.^1^). In addition to its relevance for neurodegeneration research, *Drosophila* represents an excellent experimental system for dissecting Wnt signaling dynamics, owing to the strong evolutionary conservation of this pathway. The core components of the Wnt pathway, including Wingless (Wg), Dishevelled (Dsh), Armadillo (the β-catenin homolog), frizzled 3, and Shaggy (GSK-3β homolog), are highly conserved between *Drosophila* and mammals, allowing the study of mechanistic insights across species^12,13^. In the rotenone-induced fly PD model, overactivation of the Wnt/β-catenin pathway via armadillo overexpression was shown to improve climbing performance impairment^4^, highlighting the functional relevance of this pathway in neuroprotection. Based on this, the present study aimed to examine the effect of triazine, alone or in combination with selected antioxidants, in improving symptomatic aspects of PD. We hypothesized that these combinations would exert synergistic effects, providing enhanced protection against PD pathogenesis. The results obtained support this hypothesis, providing potential new supplementation strategies to ameliorate the symptoms of PD.

## Results

### Molecular docking

**Fig. 1:**
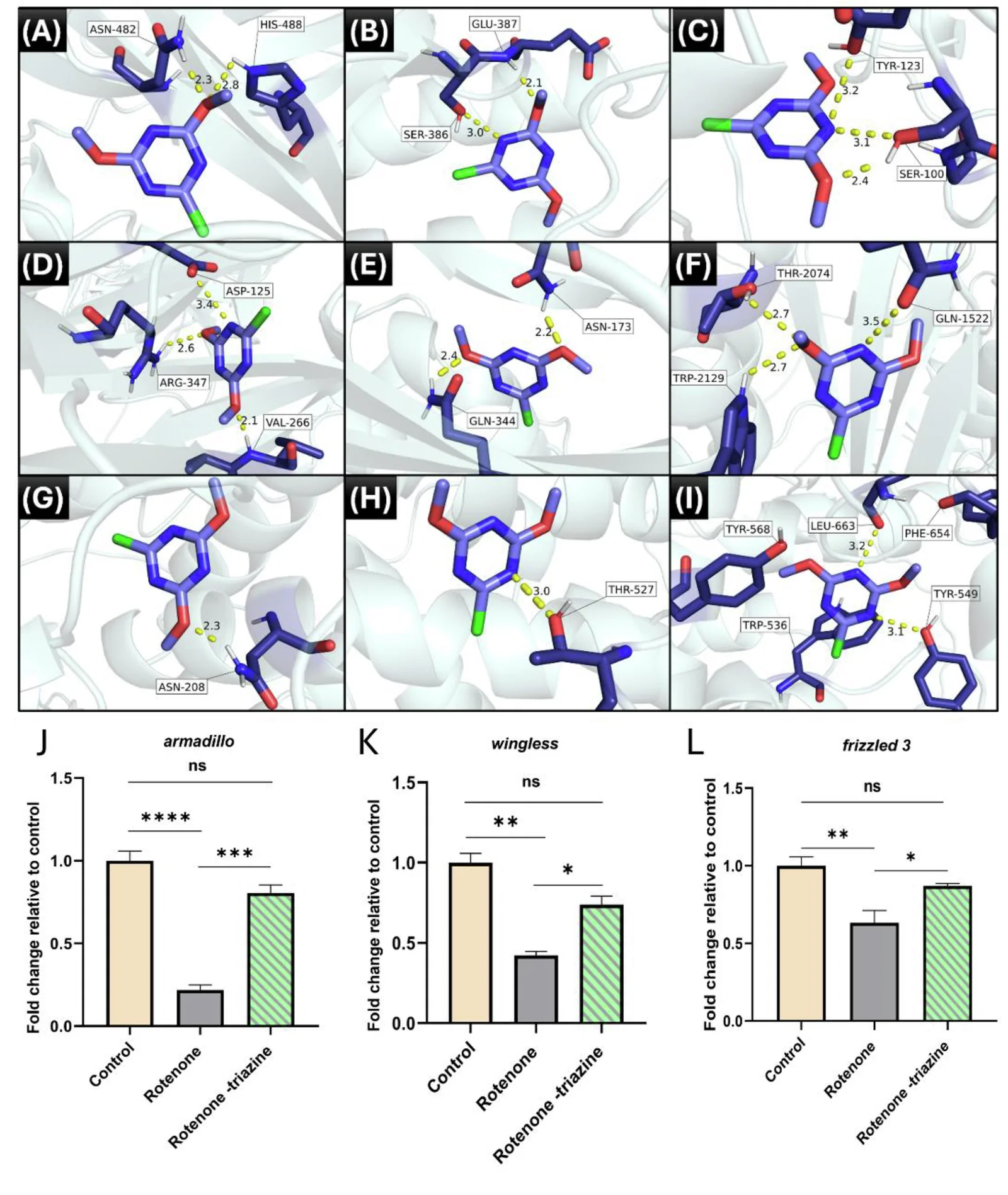
Docking poses of 2-Chloro-4,6-dimethoxy-1,3,5-triazine with the top *nine* Wnt *signaling*-related proteins. **A** Ohgata, **B** frizzled 3, **C** C-terminal Binding Protein, **D** arrow, **E** pavarotti, **F** nejire, **G** Cyclin D, **H** Adenomatous polyposis coli 2, and **I** prickle. Each panel illustrates the predicted binding orientation of the triazine *derivative* within the protein structure. Yellow dashed lines indicate predicted hydrogen bonds between the compound and key residues at the binding site. Statistical graphs of fold change in mRNA expression levels of Wnt signaling key genes, **J** *armadillo*, **K** *wingless*, and **L** *frizzled 3*, in control compared to rotenone-induced Parkinsonized flies with and without triazine supplementation. Data of qPCR are presented as mean ± SEM. Statistical analysis was performed using one-way analysis of variance (one-way ANOVA) followed by Tukey’s multiple comparisons test. Statistical significance is indicated as ns not significant; *P < 0.05, **P < 0.01; ***P < 0.001; and ****P < 0.0001.

The canonical Wnt signaling pathway plays a vital role in the maintenance of the nervous system and neuronal development^14^. The involvement of the Wnt signaling pathway has been confirmed in different neurodegenerative diseases^15–17^. Triazine derivatives were reported to activate Wnt/β catenin signaling pathway in rodent^5^ and *Drosophila*^4^ models of neurodegenerative diseases, partly due to their blood–brain barrier penetration properties, leading to increased dopamine availability, improved motor function, and neuroprotection^18^. These properties make triazine an attractive framework for developing multi-target agents capable of addressing aspects of neurodegenerative diseases^19–21^. Here, to explore proteins involved in the Wnt signaling pathway, data from the KEGG pathway (109 genes) and the AmiGO browser (140 genes) were merged, followed by removal of duplicate entries. A total of 206 unique genes were mapped to their corresponding UniProt entries and used to retrieve predicted 3D structures from the AlphaFold Protein Structure Database (Supplementary Table 1). Binding site prediction using P2Rank identified confident binding pockets (probability ≥ 0.7) in 59 proteins, which were docked specifically at the predicted sites. The remaining proteins were subjected to blind docking across the entire protein surface. Molecular docking was successfully conducted between the 2-Chloro-4,6-dimethoxy-1,3,5-triazine and 193 Wnt pathway-related proteins. Docking simulations produced binding affinity scores ranging from −5.9 to −3.4 kcal/mol (Supplementary Table 2). According to standard affinity thresholds, 71 proteins exhibited moderate binding affinity (≤−5.0 kcal/mol), suggesting a reasonable potential for interaction with it. Notably, proteins such as *ohgata* (*ohgt*)*, frizzled 3*(*fz3*)*, C-terminal Binding Protein* (*CtBP*)*, pavarotti* (*pav*), and *nejire* (*nej*) docked at predicted pockets, while *arrow* (*arr*)*, Cyclin D* (*CycD*)*, Adenomatous polyposis coli 2* (*Apc2*), and *prickle* (*pk*) docked blindly, showing some of the strongest predicted affinities within this range. Visual inspection of the docking *poses* revealed consistent ligand accommodation within the predicted or exposed binding sites, often stabilized by hydrogen bonds, particularly in the top-scoring targets (Fig. 1). Validation via quantitative rtPCR of the mRNA levels of Wnt signaling key genes demonstrated significant upregulation in the expression levels of genes armadillo, wingless, and frizzled 3 following triazine supplementation; statistical analyses revealed no significant difference between the control and Parkinsonized flies supplemented with triazine, thereby validating our in *silico* docking prediction.

### Toxicity assay of antioxidants

Based on Probit Analysis, the LC_50_ values were 1.69 mg/ mL for triazine, 2.32 mg/ mL for curcumin, and 2.01 mg/ mL for resveratrol (Table 1). The obtained LC_50_ value for each compound was then divided by 10 to determine the sublethal concentration used in all subsequent experiments, thereby ensuring minimal adverse effects of the tested compounds.

**Table 1:** The LC_50_ values of antioxidants tested in this study

Compound	LC_50_ (mg/ml)	Slope
Triazine	1.69	8.83
Curcumin	2.32	9.51
Resveratrol	2.01	7.49

### Negative geotaxis improvement in control vs rotenone-exposed flies supplemented with antioxidant alternatives

**Fig. 2:**
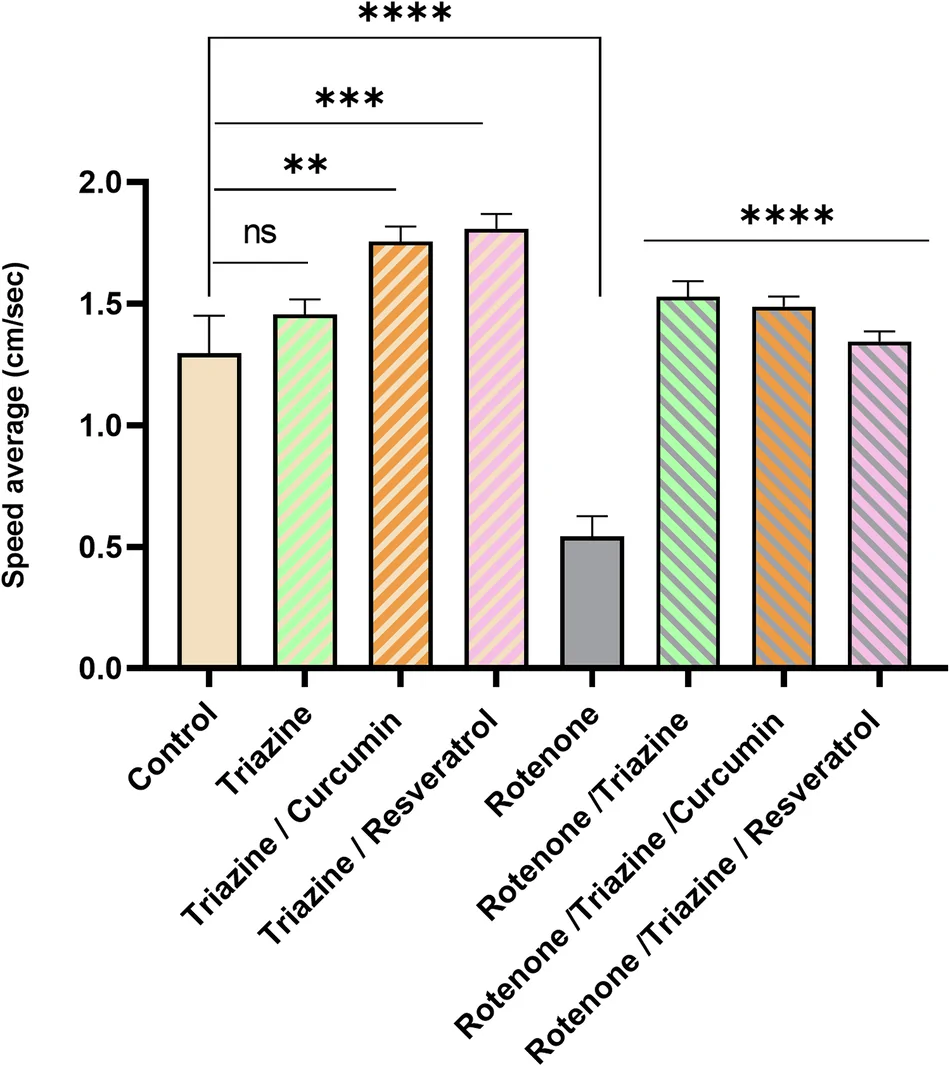
Climbing speed of adult male *Drosophila melanogaster* supplemented with triazine alone or in combination with curcumin or resveratrol at sublethal concentrations, with or without rotenone exposure. The experiment was performed in three independent biological replicates, with 10 flies per group in each replicate (*n* = 10). Data are presented as mean ± SEM. Statistical analysis was performed using one-way analysis of variance (one-way ANOVA) followed by Tukey’s multiple comparisons test. Statistical significance is indicated as ns not significant; **P < 0.01; ***P < 0.001; and ****P < 0.0001.

In preliminary trials using female flies, baseline climbing performance was highly inconsistent, yielding significant variation within the control groups even in the absence of rotenone exposure or antioxidant supplementation (Supplementary Fig. 1). Consequently, male flies were utilized for all climbing assays to ensure a reproducible baseline. To assess the improvement of locomotion activity, if any, in flies exposed to rotenone for 7 days supplemented with different antioxidants at sublethal concentrations, a negative geotaxis assay was used (Fig. 2). Results demonstrated that the climbing ability was significantly reduced (*p* < 0.0001) after rotenone exposure compared with control male flies fed permanently on normal food media. Surprisingly, no significant change was observed in the climbing ability of control flies after triazine supplementation alone, while flies supplemented with mixtures of triazine-curcumin (*p* = 0.0012) or triazine-resveratrol (*p* = 0.0002) significantly increased their movement ability compared to control. However, the ability of rotenone-exposed flies to climb improved significantly (*p* < 0.0001) with supplementation with any of the three antioxidant alternatives. Detailed statistical analysis results were provided in Supplementary Table 2.

### Dopamine Level rescue in control vs rotenone-exposed flies supplemented with antioxidant alternatives

**Fig. 3:**
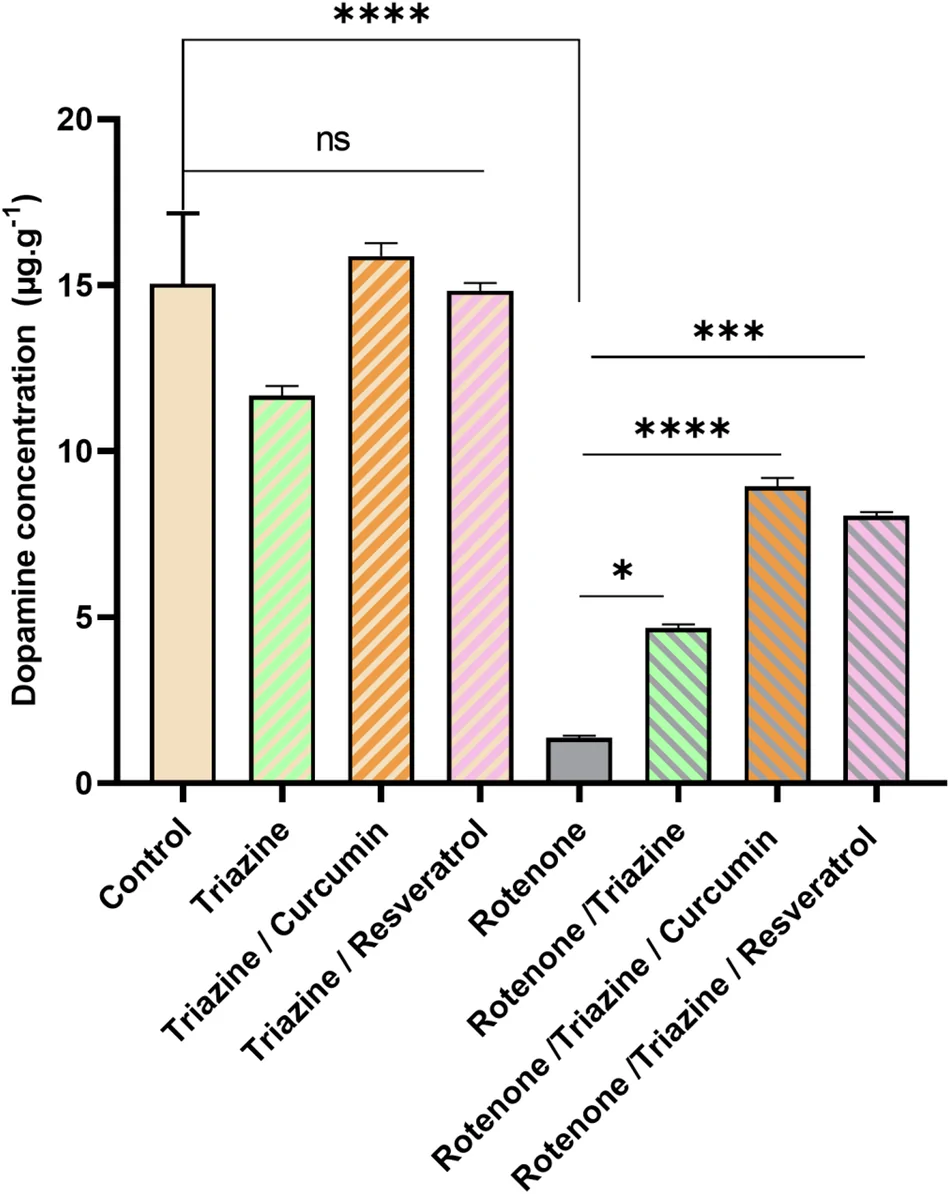
Quantification of dopamine levels in the brains of adult Drosophila melanogaster supplemented with triazine alone or in combination with curcumin or resveratrol at sublethal concentrations, with or without rotenone exposure. The experiment was performed in three independent biological replicates, with 10 flies per group in each replicate (*n* = 10). Data are presented as mean ± SEM. Statistical analysis was performed using one-way analysis of variance (one-way ANOVA) followed by Tukey’s multiple comparisons test. Statistical significance is indicated as ns not significant; *P < 0.05; ***P < 0.001; and ****P < 0.0001.

Dopamine (DA) level was quantified in control and rotenone-exposed flies either fed on normal rearing media or food media supplemented with the tested antioxidants (Fig. 3). The results revealed that DA levels were significantly reduced in rotenone-exposed flies (*p* < 0.0001) compared with the control group. No significant change was detected between control flies fed normal media and those supplemented with triazine alone or mixed with either curcumin or resveratrol (*p* > 0.5). Although triazine showed a modest but significant increase in DA level of rotenone-exposed flies, the combination of triazine and curcumin (*p* < 0.0001) or resveratrol (*p* = 0.0003) resulted in a significant increase. Detailed statistical analysis results were provided in Supplementary Table 3.

### Increase in catalase and superoxide dismutase activity in control vs rotenone-exposed flies supplemented with antioxidant alternatives

**Fig. 4:**
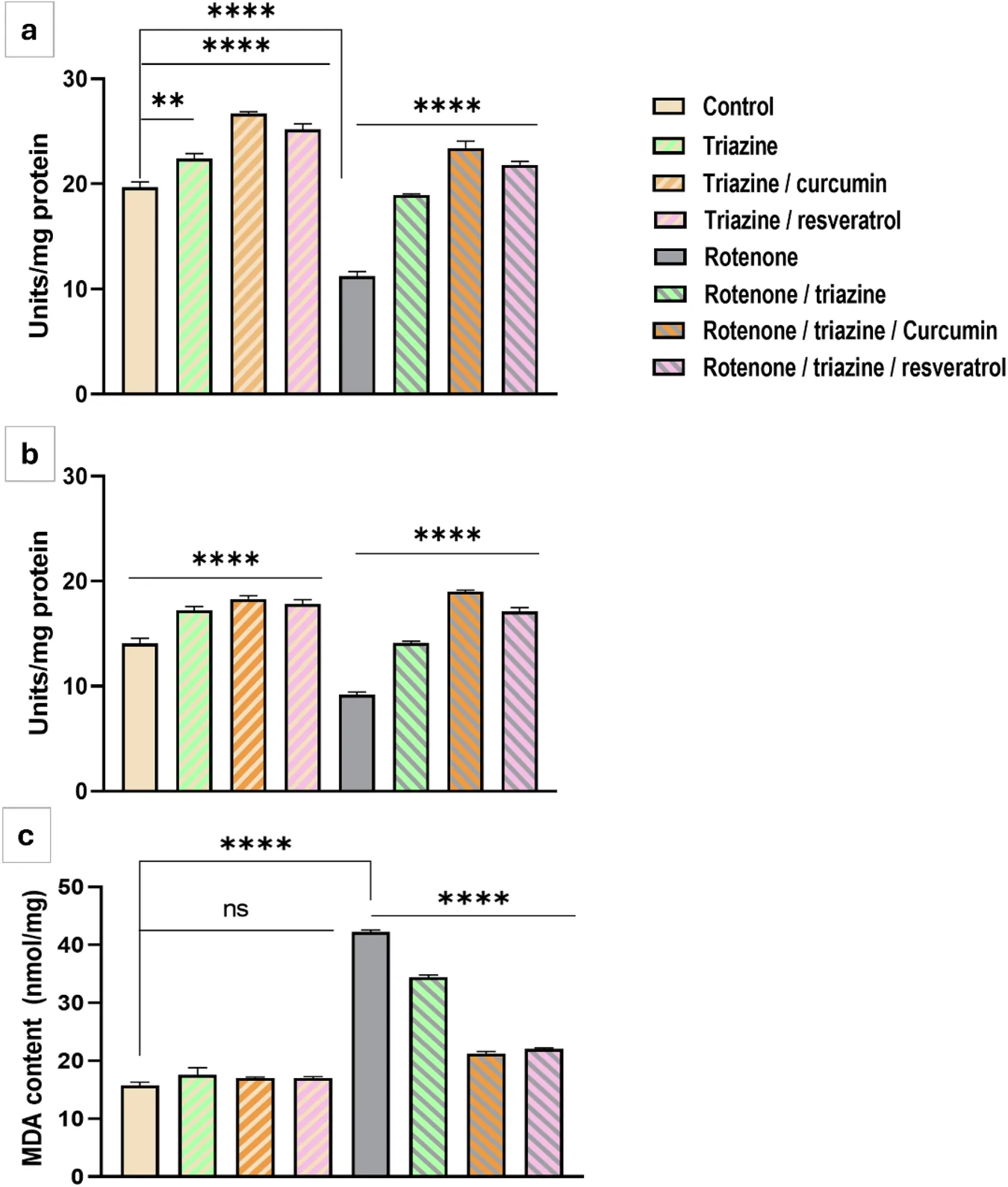
Quantification of oxidative stress and antioxidant biomarkers. Activities of catalase (**a**), superoxide dismutase (**b**) and level of MDA (**c**) were quantified in the brains of adult Drosophila melanogaster supplemented with triazine alone or in combination with curcumin or resveratrol at sublethal concentrations, with or without rotenone exposure. The experiment was performed in three independent biological replicates, with 10 flies per group in each replicate (n = 10). Data are presented as mean ± SEM. Statistical analysis was performed using one-way analysis of variance (one-way ANOVA) followed by Tukey's multiple comparisons test. Statistical significance is indicated as **P < 0.01 and ****P < 0.0001.

Results revealed that supplementation with triazine alone or in combination with curcumin or resveratrol increased CAT (Fig. 4a) and Sod (Fig. 4b) activities compared to those in control-fed flies. Significant rescue of both antioxidant enzyme activities was detected in rotenone-exposed flies supplemented with antioxidant alternatives (*P* < 0.0001) compared with flies exposed to rotenone without antioxidant supplementation. Detailed statistical analysis results were provided in Supplementary Tables 4 and 5.

### Reduction of MDA in control vs rotenone-exposed flies supplemented with antioxidant alternatives

Results revealed that MDA levels were increased in flies after rotenone exposure relative to control (*p* < 0.0001). Although triazine supplementation increased MDA in control flies, its administration alone or combined with curcumin or resveratrol significantly reduced MDA in rotenone-exposed flies (Fig. 4c). Detailed statistical analysis results were provided in Supplementary Table 6.

### Caspase-3 immunoreactivity in control vs rotenone-exposed flies supplemented with antioxidant alternatives

**Fig. 5:**
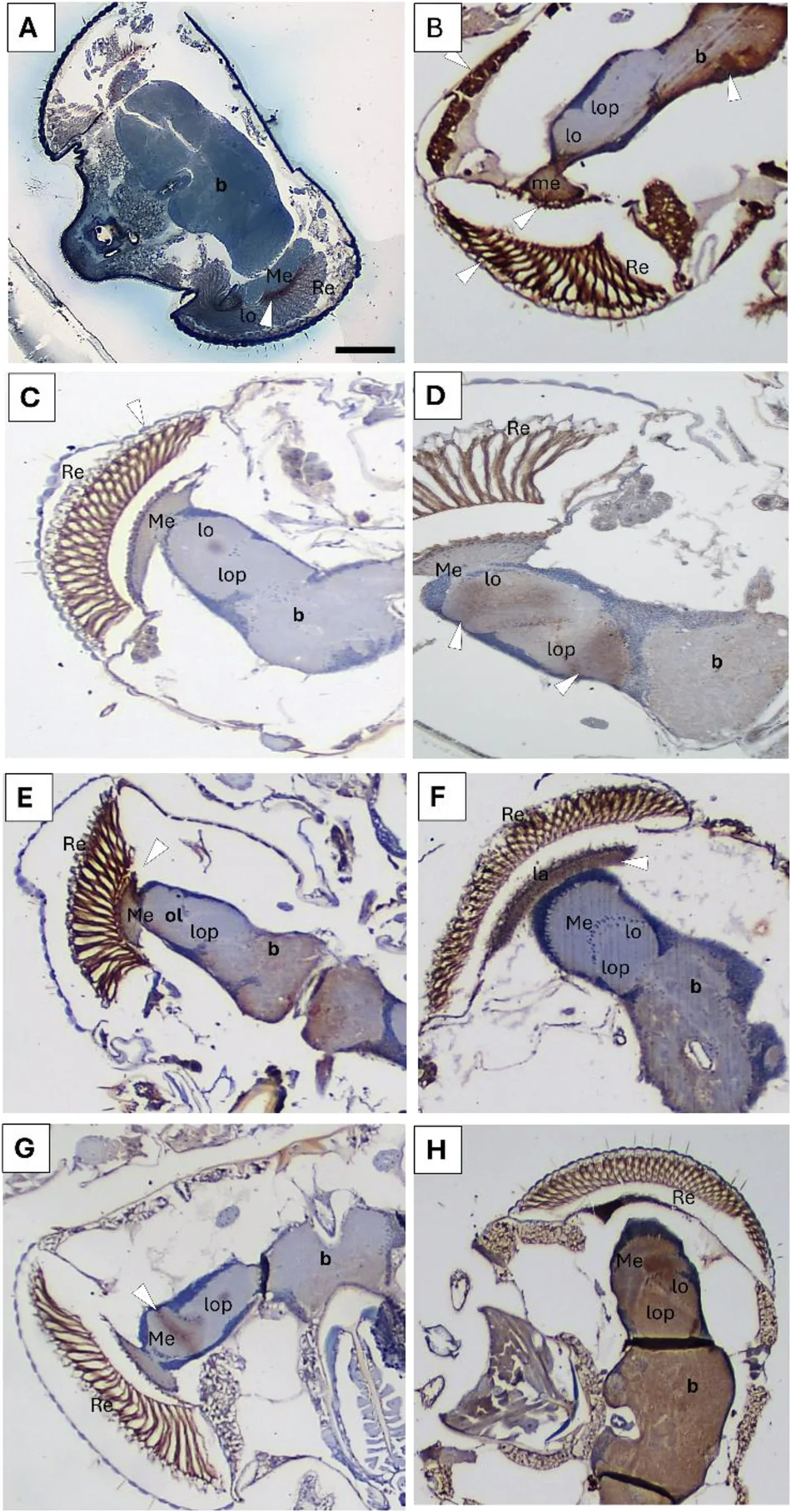
Representative transverse histological sections through the *Drosophila* head region showing immunohistochemical localization of anti-caspase-3, an apoptosis-related marker. **A** Control, **B** rotenone-exposed group, **C** triazine-supplemented group, **D** rotenone-exposed supplemented with triazine, **E** triazine-curcumin supplemented group, **F** rotenone-exposed group supplemented with triazine-curcumin, **G** triazine-resveratrol supplemented group, **H** rotenone-exposed group treated with triazine-resveratrol. Open head arrows refer to anti-caspase-3 localization; b brain, med medulla, lo lobule, lop lobular plate, Ret retina. Scale bar = 100 µm.

Results revealed that the head region of rotenone-exposed flies showed ultrastructural alterations compared with those of control flies. The tissue architecture is distorted, with evident separation and disorganization of internal structures. Areas of degeneration, vacuolization, and brain tissue shrinkage are apparent, along with disruption of normal tissue boundaries (Fig. 5). These changes indicate a clear adverse effect on tissue integrity. Abnormal accumulation of caspase-3 immunostaining within the brain and optic lobe tissues is observed. The reduction of immunostaining intensity observed in triazine (D), triazine-curcumin (F), and triazine-resveratrol (H) supplemented rotenone-exposed flies compared to their related controls without rotenone exposure (C, E, G, respectively) indicates antioxidant supplementation-associated modulation of apoptosis-related pathways. Furthermore, limited improvement was observed in healthy flies supplemented with antioxidants compared to control fed permanently on normal food media.

### Transmission electron microscopy through the head of control vs rotenone-exposed flies supplemented with antioxidant alternatives

**Fig. 6:**
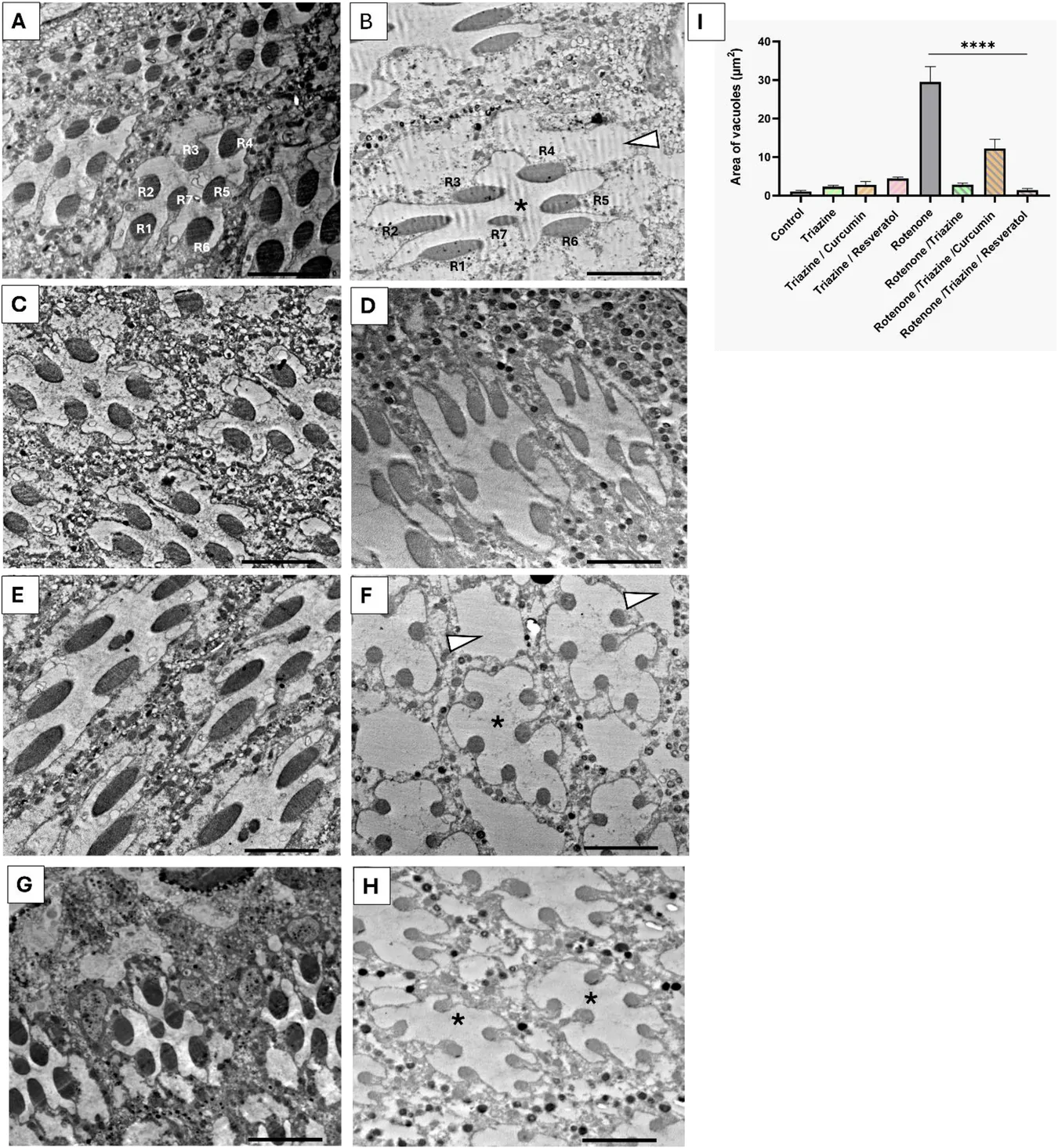
Transmission electron micrographs of compound eyes of control vs rotenone-exposed flies supplemented with antioxidant alternatives. **A** Control, **B** rotenone-exposed group, **C** triazine-supplemented group, **D** rotenone-exposed supplemented with triazine, **E** triazine-curcumin supplemented group, **F** rotenone-exposed group supplemented with triazine-curcumin, **G** triazine-resveratrol supplemented group, **H** rotenone-exposed group treated with triazine-resveratrol, and **I** statistical graph showing differences in area of vacuoles (µm^2^). R1–R7, Rhabdomeres, the eight rhabdomere not appeared as it is located beneath the seventh one. Open head arrow refers to cytoplasmic vacuolization. Bolded asterisk refers to inter-rhabdomeral space. Scale bar = 5 µm.

Ultrastructural changes arise due to rotenone exposure (Fig. 6), including compound eye structural disorganization that mirrors neurodegeneration-associated pathology, loss of the regular hexagonal ommatidial pattern, electron lucent rhabdomeres and extensive cytoplasmic vacuolization within photoreceptor cells indicating loss of cellular integrity and apoptosis. Reduced and irregular pigment granules and degeneration of pigment cells were also observed. Rotenone-exposed flies supplemented with triazine alone or combined with curcumin or resveratrol showed a marked decrease in the number of vacuoles and a statistically significant reduction in their area (*P* < 0.05), with partial restoration of cytoplasmic organization and improvement in the distribution of primary and secondary pigment cells containing pigment granules. However, the inter-rhabdomeral space occupied by the iris matrix was enlarged, indicating limited amelioration of ommatidial organization.

**Fig. 7:**
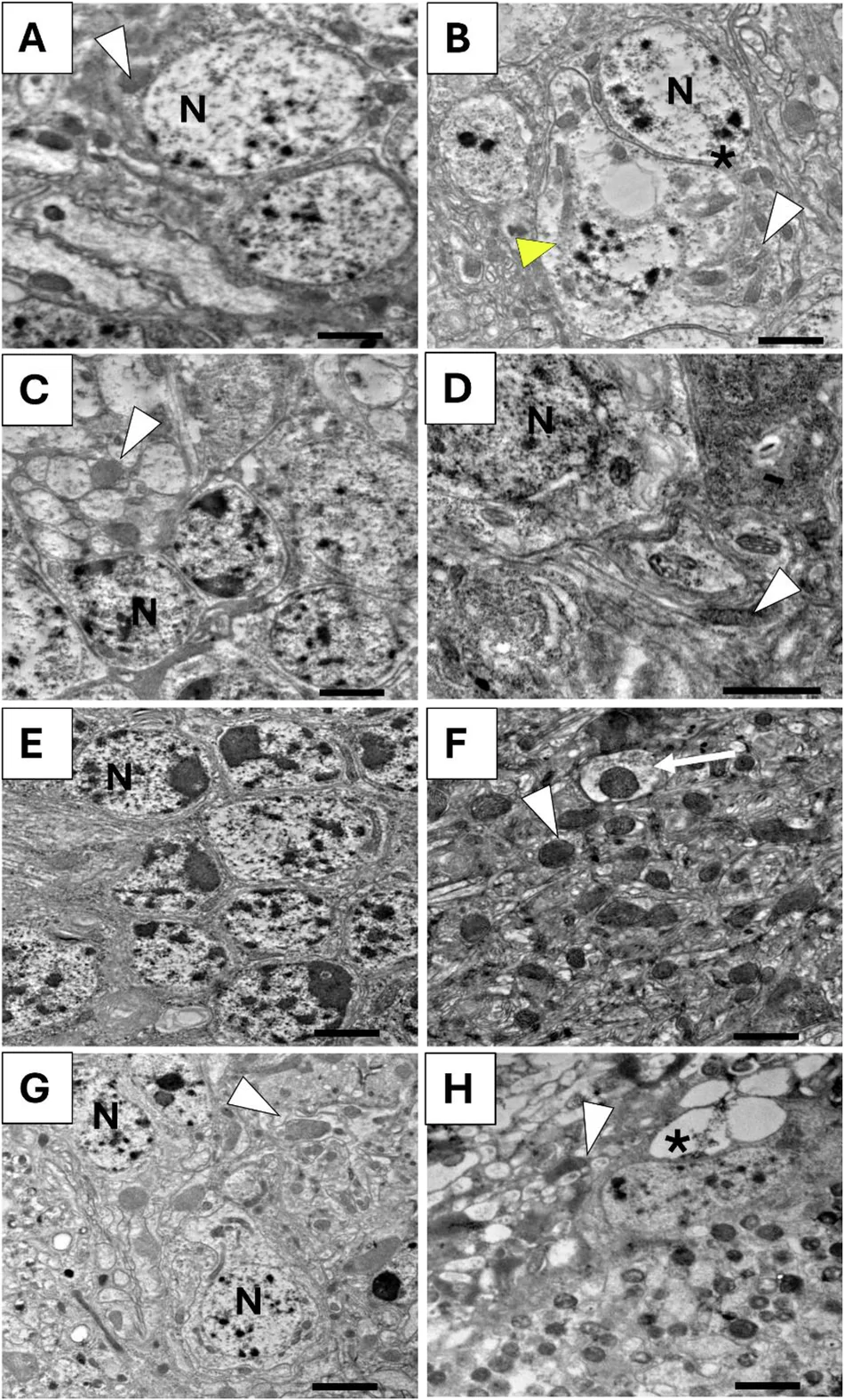
Transmission electron micrographs of brain tissue of control vs rotenone-exposed flies supplemented with antioxidant alternatives. **A** Control, **B** rotenone-exposed group, **C** triazine-supplemented group, **D** rotenone-exposed supplemented with triazine, **E** triazine-curcumin supplemented group, **F** rotenone-exposed group supplemented with triazine-curcumin, **G** triazine-resveratrol supplemented group, **H** rotenone-exposed group treated with triazine-resveratrol, N nucleus. The yellow head arrow refers to the abnormality that occurred in nuclei within the brain tissue of rotenone-exposed flies. White head arrow refers to mitochondria. Bolded asterisk refers to vacuolation. Scale bar = 1 µm.

The neural tissue of rotenone-treated flies shows structural deterioration (Fig. 7). Mitochondria appear elongated or irregular, with loss of cristae organization, which is a common indicator of mitochondrial dysfunction. The cytoplasm appeared vacuolated, indicating loss of cellular integrity and degenerative or apoptotic processes. An ameliorative effect on cellular cohesion was observed in antioxidant-supplemented rotenone-exposed flies based on the visible ultrastructural features such as less cytoplasmic vacuolization. Mitochondria of control and triazine- and triazine-antioxidant-supplemented flies (Supplementary Fig. 2A–H) showed regular morphology with intact membranes and tightly packed parallel cristae. While mitochondria of Rotenone-exposed flies appeared irregular swelling, and severely degenerating with short cristae, heterogeneous in size, indicating mitochondrial dysfunction and oxidative stress. Although some mitochondria of Parkinsonized flies supplemented with antioxidant regimes still exhibit signs of oxidative stress, a large number have begun to regain their normal morphology in terms of the organization of their internal cristae. Quantitative analysis of mitochondria indicated significant changes in mitochondrial perimeter, Feret, solidity, and shape factor in triazine- and triazine-antioxidant-supplemented Parkinsonized flies compared to those with PD receiving no supplements. Parkinsonized flies also have a lower circularity value, indicating elongated and branched mitochondria, which is significantly different from those in flies supplemented with triazine or triazine-antioxidant combinations, in which mitochondria regain their circular shape.

## Discussion

Oxidative stress is a key contributor to PD symptoms^22,23^, leading to the use of antioxidants for symptom management since the late 20th century^24^. However, excessive antioxidant supplementation can be harmful, acting as pro-oxidants or disrupting cellular signaling and increasing health risks^25^. Therefore, this study first evaluated the intrinsic toxicity of candidate antioxidants in a *Drosophila* model. Results revealed that the LC_50_ was highest for curcumin, followed by resveratrol and triazine. Although both curcumin and resveratrol are polyphenolic compounds, our findings on their differential toxicity in the *Drosophila* model were inconsistent with observations from rodent studies, in which distinct biological responses reflecting differences in relative safety or efficacy between curcumin and resveratrol have been reported^26^. While resveratrol has been reported to have neuroprotection through dual mechanisms: upregulating endogenous antioxidant pathways and promoting autophagy signaling, which facilitates clearance of damaged mitochondria and undesired protein aggregates^27,28^, curcumin can suppress the acute and chronic inflammation^29^ and has a strong capability for scavenging superoxide radicals^30^. In addition to triazine, for which our in silico tool validated interactions with the key genes of the Wnt signaling pathway implicated in PD, we therefore considered an experimental approach that combines the beneficial effects of these compounds in a disease model. In addition, our study was further motivated by previous studies demonstrating that combining nutritional supplements, including resveratrol, provides greater neuroprotection in the zebrafish model of PD than using each supplement alone, by simultaneously targeting oxidative stress, inflammation, and cell death pathways^31^.

Molecular docking is an effective approach for identifying potential molecular targets of nutraceuticals in disease management^32^. In this study, docking analysis indicated a moderate binding affinity between triazine and 193 genes involved in the Wnt signaling pathway, suggesting a possible modulatory effect on pathway activity. The interaction was stabilized by multiple hydrogen bonds with key active-site residues. The top-scoring affinities were predicted for genes, including those that were reported to be Wnt-signaling enhancers, such as *ohgata*^33^, *frizzled 3*^34^, and *arrow*^35–41^, and Wnt signaling suppressors such as *pavarotti*^42^ and *Apc2*^43–51^. Moreover, our molecular docking results revealed that C-terminal Binding Protein^52,53^ and *nejire*^54^ can enhance or inhibit Wnt signaling. The dual function of proteins as activators and repressors for the same pathway depending on environmental signals or physiological demands was reported before^55^. In the same line, *Drosophila* prickle mutants showed increased mitochondrial oxidative stress and disruption in cellular redox balance^56^. Accordingly, the predicted pathway upregulation for some targets and down-regulation for others may represent an adaptive response rather than a contradiction to the docking results, as partial inhibition of protein activity often triggers compensatory transcriptional upregulation aimed at restoring pathway balance^57^. The quantitative RT-PCR results perfectly mirrored our in silico data. Triazine supplementation significantly activated the expression of key Wnt target genes, *armadillo*, *wingless*, and *frizzled 3*, in a Parkinsonized fly model, providing a rational mechanistic basis for the neuroprotective effects detected in vivo.

Here, using rotenone-induced parkinsonism in *Drosophila*, we reported for the first time that co-supplementation of triazine with curcumin or resveratrol significantly improved locomotor performance, resulting in no statistically significant difference between the rotenone-exposed antioxidant-supplemented groups and the control group.

Restoring the level of dopamine after antioxidant supplementation in *Drosophila* models of PD has been confirmed before^1,58–63^. Our results revealed that rotenone exposure leads to a decrease in dopamine to less than one-sixth of its normal level, but dopamine levels were enhanced when PD flies were supplemented with triazine combined with curcumin or resveratrol to a greater extent than when triazine was given alone. The effectiveness of resveratrol in preserving brain dopaminergic neurons was reported in an animal model of PD^64^. However, the results confirm that triazine, whether alone or combined with other antioxidants, significantly improves motor performance; dopamine levels rise to varying degrees depending on the supplementation regime. While a decrease in DA content strongly correlates with compromised negative geotaxis, motor impairment in *Drosophila* is not exclusively governed by dopaminergic neurotransmission. Flight, walking, and climbing behaviors rely on highly integrated neural networks that also utilize non-dopaminergic circuits, including cholinergic^65^ and serotonergic^66^ pathways. Consequently, rotenone exposure may induce systemic neurotoxic stress within these parallel motor circuits, accelerating behavioral decline even before DA depletion occurs. Furthermore, potential mismatches between absolute DA levels and climbing performance can be attributed to acute endogenous compensatory mechanisms. In early stages of neurodegeneration, the *Drosophila* central nervous system may undergo homeostatic plastic adjustments, such as upregulating postsynaptic DA receptor sensitivity or altering the firing rates of surviving dopaminergic neurons to sustain baseline motor output despite ongoing neurodegeneration. Conversely, minor DA reductions paired with subtle damage to non-dopaminergic structural networks could synergistically manifest as severe motor impairment, suggesting that although dopamine deficiency remains a hallmark of this model, future studies should undertake a comprehensive assessment that reveals the disruption in interconnected motor pathways.

Our results revealed that a combination of antioxidants increases the activity of superoxide dismutase and catalase, thereby enhancing intrinsic cellular defense systems. Through modulation of gene expression and redox-sensitive signaling pathways. This adaptive response promotes long-term resilience against oxidative stress. Importantly, strengthening endogenous antioxidant capacity is particularly relevant for dopaminergic neurons and muscle cells, where chronic oxidative stress contributes to functional decline and impaired recovery.

Oxidative stress represents a system-level challenge in PD rather than a single-pathway disturbance. Consequently, therapeutic strategies focus on restoring redox balance^1^. This perspective provides the rationale for examining antioxidants in improving symptoms of PD. Results revealed a marked reduction of MDA in parkinsonized *Drosophila* co-supplemented with triazine and curcumin or resveratrol. Physiological levels of MDA are required for adaptive signaling processes, including neuroplasticity, mitochondrial biogenesis, and muscle adaptation. Excessive antioxidant activity can disrupt these processes, leading to impaired cellular responses. Redox signaling modulators help maintain the balance between protective antioxidant activity and necessary oxidative signaling, thereby supporting dopamine synthesis, receptor sensitivity, and adaptive cellular responses without inducing reductive stress.

At the cellular level, transmission electron microscopy showed that co-supplementation with triazine and either curcumin or resveratrol markedly attenuated neurodegenerative changes in parkinsonized *Drosophila*, notably preserving mitochondrial integrity. As mitochondria are the main source of cellular ROS, their protection supports ATP production and energy-dependent processes such as locomotion, neuromuscular coordination, and dopamine synthesis; restoration of normal mitochondrial size^67^, distribution^68^, and morphology^67,69^ has been detected in *Drosophila* models of PD after antioxidant administration. Our TEM analysis revealed preservation of neuronal and compound eye architecture, with a well-organized neuropil, abundant synaptic vesicles, and intact pre- and postsynaptic membranes, indicating maintained synaptic integrity and active neurotransmission^8,9^. Healthy brains displayed numerous mitochondria with intact double membranes and clear cristae, consistent with efficient energy metabolism. In contrast, severe vacuolization and mitochondrial swelling in untreated tissue reflected irreversible cellular injury. Our findings from morphometric analysis of mitochondria further support the neuroprotective effect of triazine and triazine-antioxidant-supplemented groups supplementation in *Drosophila* PD model. Feret diameter, which represents the maximum caliper distance across an object, provides a more informative descriptor than simple diameter for irregular-shaped mitochondria because it captures structural elongation and distortion^70^. The significant reduction in both perimeter and Feret diameter following triazine and triazine-antioxidant supplementation suggests attenuation of the pathological mitochondrial elongation characteristic of oxidative stress-induced injury. In contrast, mitochondria from the Parkinsonized flies exhibited significantly lower circularity, indicating the presence of irregular and branched organelles. Higher solidity of mitochondria in triazine- and triazine-antioxidant-supplemented groups indicated improved preservation of mitochondrial integrity. Furthermore, shape factors, which quantify the degree to which mitochondria deviate from a simple circular geometry^70^ were significantly reduced in triazine- and triazine-antioxidant-supplemented groups compared to untreated PD flies. Given that mitochondrial density^10^ and morphometric homogeneity^11^ correlate with neuronal activity and resistance to neurodegenerative stress, these findings indicate that either alone or in combination with curcumin and resveratrol alleviates mitochondrial structural abnormalities induced by PD induction.

Together, in addition to positioning triazine among promising therapeutic candidates, our findings indicate that combining multi-antioxidant approaches in a *Drosophila* PD model can synergistically target oxidative stress, cellular degeneration, and behavioral deficits, reflecting that combining the benefits of several different nutritional supplements in amelioration of PD symptomatic aspects may be better than using just one supplement.

## Methods

### Molecular docking

Before in vivo experiments, the interaction potential of the 2-Chloro-4,6-dimethoxy-1,3,5-triazine with Wnt signaling-involved proteins in *Drosophila* was computationally investigated. A list of genes was generated by merging data from the KEGG pathway^71–73^ dme04310 and the AmiGO^74^ browser using the ontology term GO:0016055, with the “include regulates” filter applied, and results restricted to *Drosophila melanogaster*. Duplicate entries were removed to obtain a unique set of genes for downstream structural and docking analysis. The UniProt^75^ entries corresponding to these genes were used to retrieve predicted 3D structures from the AlphaFold Protein Structure Database^76^. The tested form of triazine was retrieved from PubChem^77^ in SMILES format and converted into a 3D SDF structure using the RDKit library. Protein and ligand structures were pre-processed using mk_prepare_receptor.py and mk_prepare_ligand.py, respectively, to assign correct charges, protonation states, and format compatibility with docking software. Binding site prediction for each protein was performed using P2Rank^78^ with the AlphaFold configuration model-c AlphaFold. For proteins with predicted pockets of probability ≥0.7, site-specific docking was performed. For proteins with lower-confidence pockets, blind docking was applied across the full protein surface. All docking simulations were performed using AutoDock Vina v1.2.7^79^ with exhaustiveness set to 64. The resulting poses were visualized using PyMOL (Schrödinger, LLC). Docking scores were interpreted based on standard thresholds: ≤−4.25 kcal/mol (weak), ≤−5.0 kcal/mol (moderate), and ≤−7.0 kcal/mol (strong) binding affinity^80^.

### *Drosophila* stocks and rearing

Canton-S flies were obtained from Bloomington *Drosophila* Stock Center (#64349; Bloomington, Indiana University, USA). The flies were reared on a standardized corn-meal-agar medium^81^ and maintained under controlled laboratory conditions at 25 °C under an 8/16 light/dark photoperiod. All procedures related to the maintenance and handling of *Drosophila melanogaster* were conducted in accordance with the ethical guidelines approved by the Research Ethics Committee, Faculty of Science, Tanta University (Institutional Animal Care and Use Committee; protocol no.IACUC-SCI-TU-0355). Live flies were randomly selected for experimental assays.

### Rotenone administration

Induction of PD-like symptomatic features in flies using rotenone (Sigma-Aldrich, Deisenhofen, Germany) was performed according to Stephano et al.^4^. Briefly, 98.6 mg of Rotenone was dissolved in 5 ml DMSO to obtain a final concentration of 50 mM. Five milliliters of the stock solution were added to the food media to get a final concentration 250 μM/L. Then, 85–100 adult flies (5 days old) were fed on food media supplemented with Rotenone for 7 days to induce parkinsonism. The control group of flies at the same age was fed on food media supplemented with 0.1% DMSO.

### Antioxidants

The triazine derivative 2-Chloro-4,6-dimethoxy-1,3,5-triazine (CAS number: 3140-73-6; Thermo Fisher Scientific, USA), curcumin (CAS: 458-37-7; Lanxess, Cologne, Germany) and resveratrol (CAS: 501-36-0; Sigma-Aldrich, USA) were used in this study. The three tested antioxidants were evaluated as triazine alone or combined with curcumin or resveratrol.

### Toxicity assay of antioxidants

Antioxidant toxicity alone was tested to identify the safe concentration ranges and to ensure that subsequent treatment effects in the PD model are accurately attributed to true neuroprotection rather than toxic or adaptive mechanisms. To do this, triazine, curcumin, and resveratrol stock solutions were prepared in DMSO at a concentration 20 mg/mL. In light of a preliminary experiment, serial dilutions of 1.72, 2.26, and 2.79 mg/20 mL of food media were prepared separately for each antioxidant. Five pairs of 5-day-old adult flies were transferred into antioxidant-supplemented media. The experiment was performed in triplicate. The number of dead flies was recorded, sexed, and LC_50_ values were calculated 7 days post-exposure. Flies fed on 0.1% DMSO-supplemented food media were used as a parallel control for the experiment. The sublethal concentrations were calculated by dividing each LC_50_ value by 10^82^.

### Quantitative real-time PCR

To validate in silico prediction, changes associated with triazine supplementation were evaluated in flies’ model of PD compared to a control group by assessing the upregulation of key genes in the Wnt signaling pathway using quantitative real-time PCR. To do this, total RNA was extracted from whole-body samples of control, Rotenone-induced Parkinson, and triazine-supplemented Parkinsonized flies (*N* = 10/group) using an RNA extraction kit (Thermo Scientific) according to the manufacturer’s instructions. RT qPCR procedures were performed as previously described (El Kholy & Al Naggar, 2023). The following primers were used: *Rpl32* forward (5′-TTGGCTTCGGTTTCCGGCAAG-3′), Rpl32 reverse (5′-ATCGATCCG ACTGGTGGCGGAT-3′), *Armadillo* forward (5′-GAACCAACAGAAACGCGCTA-3′), *Armadillo* reverse (5′-ACTGCTGTGGTTGATTCGGA-3′), *Wingless* forward (5′-CGCA GAAGCTGAAGATCCAA-3′), *Wingless* reverse (5′-GGCATCACATTGCTAGTGGT-3′), *Frizzled* forward (5′-TGATTGAGTTCTCCGCACGA-3′), *Frizzled* reverse (5′-TGCCGA AGGTGGTCAGAAAG-3′), Abundance of transcripts was quantified using the 2^-∆∆CT method. This experiment was conducted in triplicate.

### Negative geotaxis

Initial evaluations of females (*N* = 40; 10/group) climbing performance indicated substantial baseline variations across control groups prior to treatment (Fig. 1S). These pre-existing differences obscured the ability to independently assess the true impact of antioxidant supplementation on climbing performance. Therefore, male flies were chosen to test climbing performance, to avoid data variability due to female’s weight changes depending on the stage of ovulation/oogenesis^4,83^ according to Seada et al. ^84^, with minor modifications. Briefly, male flies (*n* = 10; 5-day-old) were fed on rotenone-supplemented food media for 7 days to induce parkinsonism. This specific timepoint was selected based on preliminary optimization studies to maximize the detection of robust locomotor deficits while minimizing confounding mortality^85,86^. Extending rotenone exposure beyond 7 days resulted in accelerated mortality rates, which would introduce survivorship bias into the behavioral readouts. After rotenone exposure, flies were transferred to vials containing either control or antioxidant-supplemented food media. After 7 days, the climbing ability of the flies was assessed by transferring them into an empty 100 mL glass cylinder. After 10 min acclimation at room temperature, the cylinder was gently tapped to the bottom, and the upward movement of flies to the top of the cylinder was videotaped for 2 min. Speed of climbing (cm/s) was calculated using frame-by-frame analysis in ImageJ software (version 1.2)^87^. The same number of males fed for 7 days on normal food media, followed by feeding for 7 days on antioxidant-supplemented food media at sublethal concentration as mentioned above, was used as a positive control for the whole experiment. A parallel negative control was kept permanently on 0.1% DMSO-supplemented food media.

### Determination of dopamine level using high-performance liquid chromatography (HPLC)

Dopamine level was quantified according to Hakim et al.^88^. Briefly, fly’s heads (15/exposure; 3 replica/exposure) were dissected, homogenized, and sonicated in 200 µl of 0.1 M phosphate-buffered saline. The homogenates were centrifuged at 6000 rpm for 10 min at 4 °C, and 100 µl of the supernatant was mixed with 100 µl of 5% trichloroacetic acid. Samples were stored at −80 °C until further analysis. Dopamine concentration was determined using an HPLC system (E Chromatic model LC 1620) equipped with a C18 column and a UV detector set at 280 nm. The mobile phase consisted of water: acetonitrile (9:1, v/v) delivered at a flow rate of 1 ml/min. The Injection Volume Was 25 µl, And Data Acquisition And Processing Were Performed Using Chemstation version 2.0 and PA Station 2015 software.

### Biochemical parameters

Fly heads from each group were dissected (50 heads/exposure; three replica/exposure) and homogenized in 0.1 M phosphate buffer. After centrifugation, the supernatant was aliquoted and kept at −80 °C for performing subsequent assays.

The Catalase activity (CAT) was assessed using a BT Lab assay kit (#SH0023; Zhejiang, China) according to the manufacturer’s protocol. Absorbance was measured at 405 nm.

Immunoassay quantification of Superoxide dismutase (SOD) was performed using a BT Lab assay kit (#SH0039; Zhejiang, China) according to manufacturer instructions in a 96-well Microplate. Absorbance was measured at 450 nm wavelength.

Colorimetric assay of lipid peroxidation was performed using MDA Assay Kit (MAK085; Sigma-Aldrich, USA) according to the manufacturer’s instructions. Spectrophotometric measurement was performed at 532 nm wavelength.

### Immunohistochemistry anti-caspase-3

To assess neuronal apoptosis, immunohistochemical staining was conducted using an anti-cleaved Caspase-3 antibody. After being fixed in 10% neutral-buffered formalin, ten *Drosophila* heads were dehydrated using a graded series of ethanol. Following the embedding and sectioning of fixed tissues, sections were deparaffinized and rehydrated, washed twice, followed by blocking nonspecific background by incubating sections in Hydrogen peroxide blocking buffer. The sections were then washed and stained using anti-Caspase-3 antibody (GB11532, Servicebio Technology, Wuhan, Hubei, China) at a dilution of 1:200. Following washing, the sections were left at room temperature for 30 min to incubate with HRP-conjugated secondary antibody (ScyTek Laboratories, Logan, Utah, USA). A brown coloration, signifying positive staining, was then observed using 3,3′-diaminobenzidine tetrahydrochloride (DAB) chromogen solution after sections had been treated with streptavidin for 10 min. Hematoxylin was used to counterstain the slides, which were then extensively cleaned and dehydrated using increasing alcohol concentrations (70%, 80%, 90%, and 100%) and cleansed in xylene for 5 min. Tonsil tissue was used as a positive control in every staining batch. To evaluate immunoreactivity, the stained slices were lastly mounted and viewed under a Leitz–Wetzlar photomicroscope (modified from Huang et al.^89^).

### TEM

The heads of the flies (*N* = 10) were dissected, fixed in a 2.5–3% glutaraldehyde solution prepared in 0.1 M cacodylate buffer (pH 7.2–7.4) at 4 °C for 2 h. Post-fixation was carried out using 1% osmium tetroxide at 4 °C for an additional 1.5 h. The specimens were then dehydrated through a graded ethanol series (50%, 70%, 90%, 95%, and four changes of 100%), with each step lasting 15 min, followed by a 30-min dehydration step in acetone. Subsequently, the samples were embedded in epoxy resin using the Epoxy Embedding Medium Kit (Sigma, Germany). Ultra-thin sections were prepared using an RMC PT-XL PowerTome Ultramicrotome, producing both semi-thin and ultra-thin slices. Ultra-thin sections, 70–90 nm thick, were stained with 2.5% uranyl acetate as the primary stain and counterstained with lead citrate, examined and imaged using a JEM-2100 transmission electron microscope (JEOL, Japan). Quantitative assessment of mitochondrial morphology was performed on TEM images using Fiji/imagej software. Prior to measurement, the spatial scale of each TEM image was calibrated in Fiji using the scale bar provided on the micrograph. Images were selected from comparable anatomical regions and at comparable magnification across experimental groups; three images from three independent biological samples were analyzed. Mitochondria that were severely visible were excluded from morphometric analysis. The outer boundary of each selected mitochondrion was manually traced using the freehand tool in Fiji/ImageJ. The enclosed region was then used to determine mitochondrial area, perimeter, Feret diameter, circularity, solidity, and shape factor. Circularity was calculated by Fiji/imagej according to the equation 4π × Area/Perimeter^2^, where a value of 1 represents a perfect circle and lower values indicate elongated or irregular shapes. Solidity was calculated as the ratio of mitochondrial area to the area of its convex hull, with lower values indicating boundary irregularity. The shape factor parameter was estimated as previously described^90^, with a value of 1 for a perfect circle. The same image analysis procedure was applied consistently across all experimental groups.

### Statistical analysis

All data are presented as mean ± standard error of the mean (SEM). Data normality was assessed using the Shapiro–Wilk test in Minitab software (version 20.4). All datasets met the normality assumption except for the mitochondrial assay data. Logarithmic transformation was applied to the mitochondrial assay data; however, the data remained non-normally distributed. Therefore, the mitochondrial assay data were analyzed using the Kruskal–Wallis test followed by Dunn’s multiple comparisons post hoc test. All other datasets were analyzed using one-way analysis of variance (ANOVA) followed by Tukey’s multiple comparisons post hoc test. Statistical analyses were performed using GraphPad Prism (version 8.0), and a *P* value < 0.05 was considered statistically significant.

## Supplementary information

Supplementary Information

## Data Availability

The datasets are available in supplementary files.
