# Supplementary material for: Synergistic interplay between triazine and curcumin or resveratrol in mitigating rotenone-induced toxicity in Drosophila model
Source: NPJ Parkinsons Dis. 2026 Nov 11;12:278. doi: 10.1038/s41531-026-01592-5 (PMC13754896; doi:10.1038/s41531-026-01592-5)
Supplement: Supplementary file 1 [file 41531_2026_1592_MOESM1_ESM.pdf]

Supplementary Table 1: Affinity data of 206 unique genes mapped to their corresponding UniProt entries and used to retrieve predicted 3D structures from the AlphaFold Protein Structure Database.

Gene_Symbol	Protein	Affinity
**ohgt**	Q9VH36	-5.913
**fz3**	O77438	-5.871
**CtBP**	O46036	-5.758
**arr**	A1Z9D7	-5.718
**pav**	Q9VZF5	-5.679
**nej**	M9MS40	-5.573
**CycD**	Q7KUZ5	-5.571
**Apc2**	Q9Y1T2	-5.57
**pk**	A1Z6W3	-5.56
**Galphao**	P16378	-5.553
**Wnt10**	Q9VM25	-5.543
**Smox**	O96660	-5.513
**sfl**	Q9V3L1	-5.454
**Wnk**	M9PGC5	-5.422
**Sirt1**	Q9VK34	-5.414
**fz2**	Q9VVX3	-5.412
**Pp2B-14D**	Q27889	-5.369
**Pp1alpha-96A**	P48461	-5.358
**Rnf146**	B7Z078	-5.327
**sona**	B7YZP6	-5.324
**tum**	A1Z9I5	-5.323
**CkIIalpha**	P08181	-5.286
**hpo**	Q8T0S6	-5.269
**SkpA**	O77430	-5.268
**Wnt6**	Q9VM26	-5.267
**CaMKII**	Q00168	-5.266
**kermit**	Q7JX82	-5.247
**CanA1**	P48456	-5.243
**pont**	Q9VH07	-5.241
**Reck**	Q9VUJ2	-5.234
**DAAM**	M9MSM5	-5.203
**Ssdp**	A0A0B4KGW6	-5.193
**inaC**	P13677	-5.193
**osa**	Q8IN94	-5.176
**gro**	P16371	-5.163
**por**	Q9VWV9	-5.16
**hyd**	P51592	-5.156

**Dyrk2**	Q9V3D5	-5.152
**Vang**	A0A0B4KEN4	-5.152
**wntD**	Q9VFX1	-5.149
**CanA-14F**	Q9VXF1	-5.147
**Rho1**	P48148	-5.147
**Tak1**	Q9V3Q6	-5.14
**CG9962**	Q9VQE3	-5.137
**Cul6**	Q9VU33	-5.109
**Notum**	Q9VUX3	-5.093
**Sin3A**	A0A0B4K765	-5.092
**kto**	Q9VW47	-5.092
**Ste:CG33237**	Q7KV13	-5.088
**Pp1-87B**	P12982	-5.088
**Usp8**	Q9VDD8	-5.077
**norpA**	P13217	-5.073
**Wnt5**	P28466	-5.071
**rept**	Q9V3K3	-5.07
**Cdc16**	Q9VCN6	-5.062
**mts**	P23696	-5.062
**Tnks**	Q9VBP3	-5.061
**Oseg4**	Q9W097	-5.058
**dlp**	Q9VUG1	-5.05
**Klp64D**	Q9VRK9	-5.043
**Eip63E**	M9PE01	-5.036
**CkIalpha**	P54367	-5.036
**rk**	Q7KTA0	-5.028
**CG2577**	Q9VYN5	-5.021
**Takl1**	P83104	-5.011
**wg**	P09615	-5.009
**wts**	Q9VA38	-5.007
**Wnt2**	P28465	-4.994
**Cow**	Q9VCR3	-4.983
**flw**	P48462	-4.963
**Oseg1**	Q9VSJ6	-4.953
**Apc**	Q9VAS9	-4.916
**Hipk**	Q9W0Q1	-4.909
**lic**	O62602	-4.898
**sgg**	P18431	-4.897
**CG6420**	Q9VBC4	-4.894
**drl**	Q27324	-4.894
**nkd**	Q9VVV9	-4.879

**Kap3**	Q9VZ07	-4.877
**Pka-C1**	P12370	-4.873
**TER94**	Q7KN62	-4.867
**Bili**	Q9VBX0	-4.854
**ATP6AP2**	Q9VHG4	-4.852
**nito**	Q7KMJ6	-4.847
**shf**	Q9W3W5	-4.836
**fz**	P18537	-4.834
**sinah**	Q8T3Y0	-4.833
**Mmp2**	Q8MPP3	-4.829
**p120ctn**	Q7PLI0	-4.821
**dgo**	Q7JUF2	-4.819
**Wnt4**	P40589	-4.819
**gskt**	P83101	-4.819
**CG9062**	Q1LZ08	-4.816
**nord**	Q9W192	-4.809
**tws**	P36872	-4.805
**rempA**	Q7KTZ4	-4.801
**dx**	Q23985	-4.797
**Drl-2**	Q7JQT0	-4.789
**Usp47**	Q24574	-4.788
**skd**	Q7KTX8	-4.774
**gish**	A0A0B4KHL4	-4.764
**Takl2**	Q9VCV0	-4.757
**dally**	Q24114	-4.753
**Vps35**	Q9W277	-4.745
**arm**	P18824	-4.744
**Mtl**	Q7JWS8	-4.742
**CG12147**	Q7JWT4	-4.741
**Lmpt**	Q7KUQ6	-4.738
**wls**	Q95ST2	-4.73
**dnt**	Q9V422	-4.729
**Pka-C2**	P16911	-4.724
**Cul1**	Q24311	-4.719
**Prosap**	A1Z9K8	-4.719
**Pkc53E**	P05130	-4.718
**pbl**	Q8IQ97	-4.718
**Swim**	Q7JWQ7	-4.711
**dsh**	P51140	-4.711
**gig**	Q9VW83	-4.705
**Rac2**	P48554	-4.693

**Ste:CG33236**	Q7KV12	-4.688
**Rac1**	P40792	-4.685
**Ste:CG33246**	Q7KV22	-4.657
**bsk**	P92208	-4.656
**RhoL**	Q24192	-4.65
**Diap1**	Q24306	-4.649
**Ssl**	Q24536	-4.636
**dco**	O76324	-4.634
**CG34404**	Q9VF73	-4.633
**Ste:CG33239**	Q7KV15	-4.632
**trbd**	Q9VH90	-4.63
**nmo**	A0A4D6K823	-4.611
**Ste:CG33242**	Q7KV18	-4.596
**pan**	Q8IMA8	-4.587
**Duba**	Q9VTK7	-4.584
**otk2**	Q7JXW8	-4.576
**apolpp**	Q9V496	-4.566
**Plc21C**	P25455	-4.551
**Nulp1**	Q9Y109	-4.548
**fz4**	Q9NBW1	-4.539
**Sulf1**	Q9VEX0	-4.538
**SkpB**	Q7KJ69	-4.516
**hyx**	Q9VHI1	-4.514
**otk**	Q6AWJ9	-4.511
**Nek2**	Q9W3N8	-4.507
**Ste:CG33238**	Q7KV14	-4.505
**aop**	Q01842	-4.502
**CG15365**	X2JD14	-4.477
**Ste:CG33247**	Q7KV23	-4.475
**CG15800**	Q9W1J7	-4.47
**crol**	O61360	-4.469
**sina**	P21461	-4.468
**SkpD**	Q9VWC5	-4.467
**lgs**	Q961D9	-4.459
**slmb**	A0A0B4KHK1	-4.451
**Coop**	Q7JUZ9	-4.444
**Vps26**	Q9W552	-4.442
**CkIIbeta2**	O96863	-4.422
**Ror**	Q24488	-4.417
**Gint3**	Q7K0S5	-4.412
**CycY**	Q9VKF0	-4.406

**CG43089**	M9ND39	-4.385
**RpS12**	P80455	-4.38
**SkpE**	Q9VWC3	-4.371
**Ste:CG33243**	Q7KV19	-4.368
**ewg**	Q24312	-4.36
**Usp12-46**	Q9VCT9	-4.358
**CkIIbeta**	P08182	-4.34
**CG7094**	Q9VJC2	-4.326
**Ste12DOR**	Q9NIV2	-4.282
**esn**	Q9U1I1	-4.282
**SkpF**	Q9W1Q0	-4.275
**CG3226**	Q9W3Y3	-4.26
**phyl**	Q27934	-4.258
**SkpC**	Q9VWC4	-4.241
**ebd1**	Q9W0N9	-4.231
**Axn**	Q9V407	-4.19
**CG11523**	Q9VNV2	-4.169
**ebi**	Q95RJ9	-4.146
**Psn**	O02194	-4.116
**SoxN**	M9PCZ7	-4.072
**CanB**	P48451	-4.036
**pygo**	Q9V9W8	-4.012
**Roc1b**	Q9NHX0	-4.004
**CanB2**	Q24214	-3.993
**CG17278**	Q9VDG1	-3.885
**Myc**	Q9W4S7	-3.873
**Roc1a**	Q9W5E1	-3.853
**CG14505**	Q9V8F3	-3.783
**IFT43**	Q9VK67	-3.745
**RanBP3**	Q9VCI7	-3.701
**tsh**	P22265	-3.644
**Jra**	P18289	-3.549
**Cby**	B9A0M5	-3.414

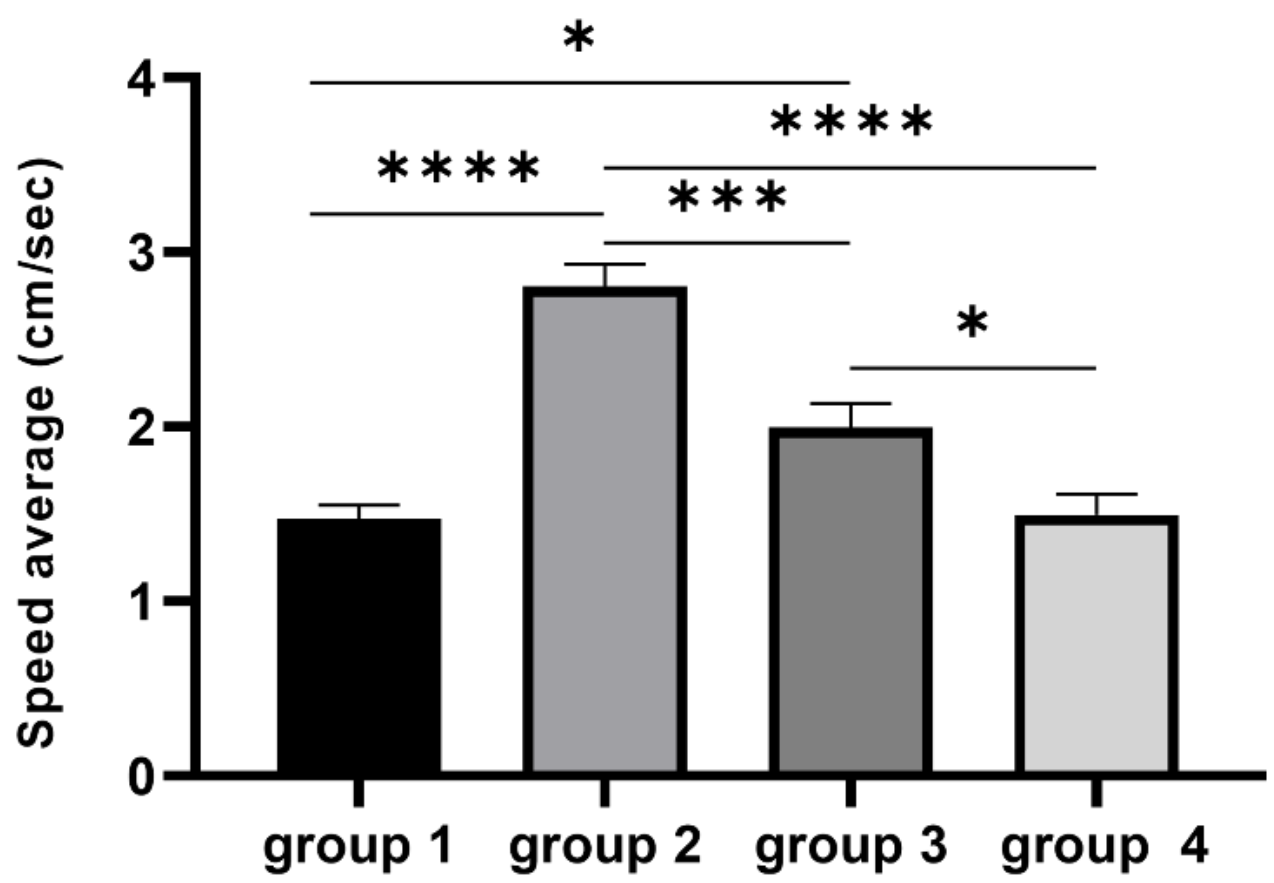

Supplementary Figure 1: Climbing Speed of female (N=40; 10/group) *Drosophila*. Data are presented as Mean ± SEM; Statistical significance (One-way ANOVA) is indicated as ns, not significant; *$P < 0.05$; ***$P < 0.001$; and ****$P < 0.0001$.

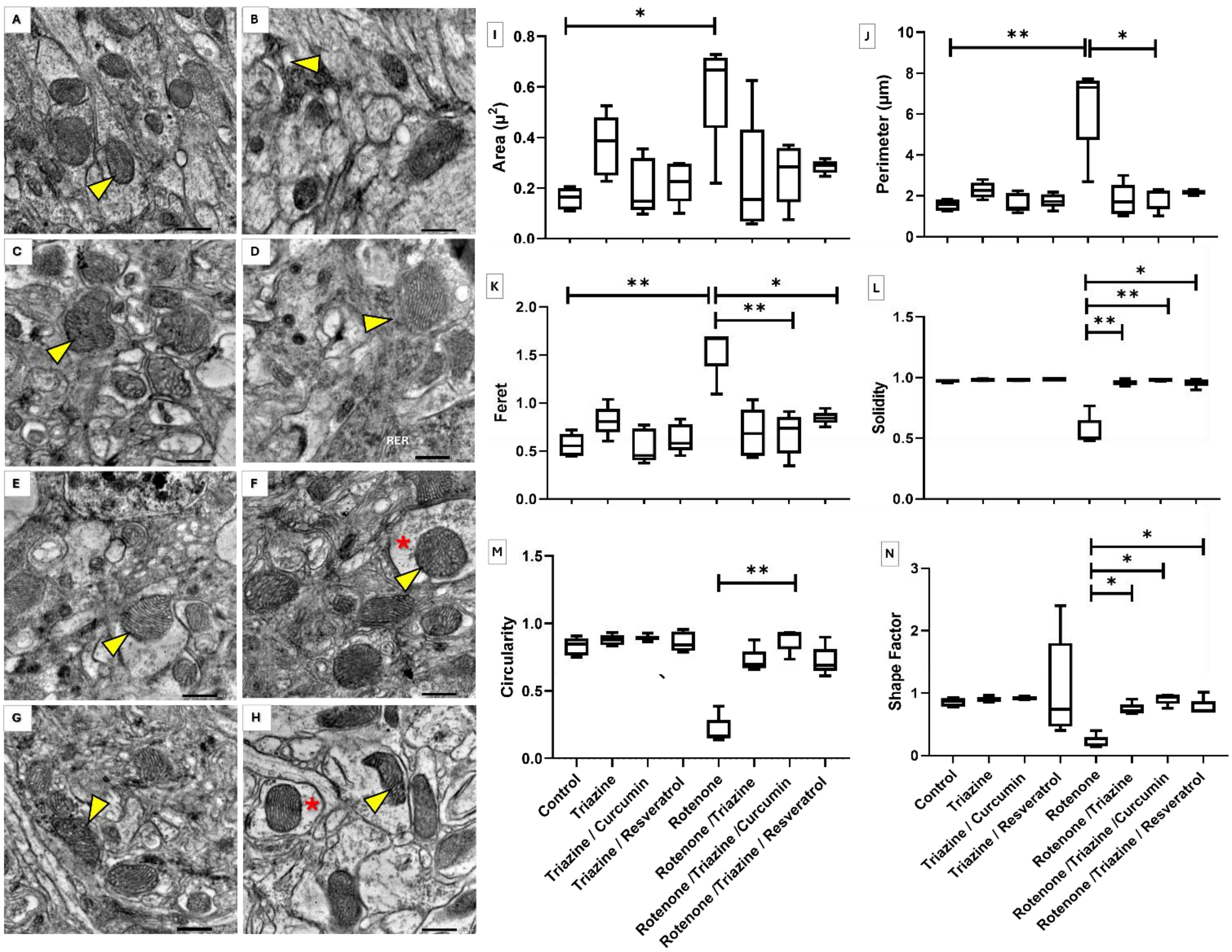

Supplementary Figure 2: Transmission electron micrographs showed mitochondrial morphology in control vs rotenone-exposed flies supplemented with antioxidants alternatives. A, control, B, rotenone-exposed group, C, triazine supplemented group, D, rotenone-exposed supplemented with triazine, E, Curcumin-supplemented group, F, rotenone-exposed group supplemented with curcumin, G, resveratrol-supplemented group, H, rotenone-exposed group treated with resveratrol. Yellow head arrow refers to mitochondria; red asterisk refers to vacuoles and RER refers to rough endoplasmic reticulum. Scale bar = 5 µm. Sub-figures I-N, illustrated the quantitative changes in mitochondrial area, perimeter, Feret, solidity, circularity and shape factor, respectively. Data is presented as medians with interquartile range. Statistical significant was determined using Kruskal-Wallis's test followed by Dunn's Post-hoc test for multiple comparison. * $P < 0.05$ and ** $P < 0.01$.

Supplementary Table 2: p-values and 95% confidence intervals (CI) for mean differences calculated for all pairwise comparisons for Climbing performance (negative geotaxis) in adult male *Drosophila melanogaster* receiving either triazine alone or in combination with curcumin or resveratrol (± rotenone exposure) at sub-lethal concentrations.

**Tukey's multiple comparisons test**	**95.00% CI of diff.**	**Adjusted P Value**
Control vs. Triazine	-0.5005 to 0.1787	0.8353
Control vs. Triazine / Curcumin	-0.7997 to -0.1205	0.0012
Control vs. Triazine / Resveratrol	-0.8524 to -0.1732	0.0002
Control vs. Rotenone	0.4131 to 1.092	<0.0001
Control vs. Rotenone /Triazine	-0.5737 to 0.1055	0.4151
Control vs. Rotenone /Triazine /Curcumin	-0.5313 to 0.1479	0.6729
Control vs. Triazine / Resveratrol	-0.3883 to 0.2909	0.9999
Triazine vs. Triazine / Curcumin	-0.6388 to 0.04038	0.1300
Triazine vs. Triazine / Resveratrol	-0.6915 to -0.01234	0.0361
Triazine vs. Rotenone	0.5740 to 1.253	<0.0001
Triazine vs. Rotenone /Triazine	-0.4128 to 0.2664	0.9980
Triazine vs. Rotenone /Triazine /Curcumin	-0.3704 to 0.3088	>0.9999
Triazine vs. Triazine / Resveratrol	-0.2274 to 0.4518	0.9731
Triazine / Curcumin vs. Triazine / Resveratrol	-0.3923 to 0.2869	0.9998
Triazine / Curcumin vs. Rotenone	0.8732 to 1.552	<0.0001
Triazine / Curcumin vs. Rotenone /Triazine	-0.1136 to 0.5656	0.4631
Triazine / Curcumin vs. Rotenone /Triazine /Curcumin	-0.07116 to 0.6080	0.2393
Triazine / Curcumin vs. Triazine / Resveratrol	0.07181 to 0.7510	0.0062
Triazine / Resveratrol vs. Rotenone	0.9259 to 1.605	<0.0001
Triazine / Resveratrol vs. Rotenone /Triazine	-0.06088 to 0.6183	0.1975
Triazine / Resveratrol vs. Rotenone /Triazine /Curcumin	-0.01845 to 0.6607	0.0790
Triazine / Resveratrol vs. Triazine / Resveratrol	0.1245 to 0.8037	0.0010
Rotenone vs. Rotenone /Triazine	-1.326 to -0.6472	<0.0001
Rotenone vs. Rotenone /Triazine /Curcumin	-1.284 to -0.6047	<0.0001
Rotenone vs. Triazine / Resveratrol	-1.141 to -0.4618	<0.0001
Rotenone /Triazine vs. Rotenone /Triazine /Curcumin	-0.2972 to 0.3820	>0.9999
Rotenone /Triazine vs. Triazine / Resveratrol	-0.1542 to 0.5250	0.7095
Rotenone /Triazine /Curcumin vs. Triazine / Resveratrol	-0.1966 to 0.4826	0.9042

Supplementary Table 3: p-values and 95% confidence intervals (CI) for mean differences calculated for all pairwise comparisons of quantification of dopamine in brains of adult flies supplemented with supplemented with triazine alone or in combination with curcumin or resveratrol (± rotenone exposure) at sub-lethal concentrations.

Tukey's multiple comparisons test	95.00% CI of diff.	Adjusted P Value
Control vs. Triazine	-0.4577 to 7.191	0.1064
Control vs. Triazine / Curcumin	-4.664 to 2.984	0.9931
Control vs. Triazine / Resveratrol	-3.614 to 4.034	>0.9999
Control vs. Rotenone	9.849 to 17.50	<0.0001
Control vs. Rotenone /Triazine	6.542 to 14.19	<0.0001
Control vs. Rotenone /Triazine / Curcumin	2.286 to 9.934	0.0009
Control vs. Triazine / Resveratrol	3.156 to 10.80	0.0002
Triazine vs. Triazine / Curcumin	-8.031 to -0.3823	0.0259
Triazine vs. Triazine / Resveratrol	-6.981 to 0.6677	0.1478
Triazine vs. Rotenone	6.482 to 14.13	<0.0001
Triazine vs. Rotenone /Triazine	3.176 to 10.82	0.0002
Triazine vs. Rotenone /Triazine / Curcumin	-1.081 to 6.568	0.2689
Triazine vs. Triazine / Resveratrol	-0.2110 to 7.438	0.0712
Triazine / Curcumin vs. Triazine / Resveratrol	-2.774 to 4.874	0.9754
Triazine / Curcumin vs. Rotenone	10.69 to 18.34	<0.0001
Triazine / Curcumin vs. Rotenone /Triazine	7.382 to 15.03	<0.0001
Triazine / Curcumin vs. Rotenone /Triazine / Curcumin	3.126 to 10.77	0.0002
Triazine / Curcumin vs. Triazine / Resveratrol	3.996 to 11.64	<0.0001
Triazine / Resveratrol vs. Rotenone	9.639 to 17.29	<0.0001
Triazine / Resveratrol vs. Rotenone /Triazine	6.332 to 13.98	<0.0001
Triazine / Resveratrol vs. Rotenone /Triazine / Curcumin	2.076 to 9.724	0.0013
Triazine / Resveratrol vs. Triazine / Resveratrol	2.946 to 10.59	0.0003
Rotenone vs. Rotenone /Triazine	-7.131 to 0.5177	0.1170
Rotenone vs. Rotenone /Triazine / Curcumin	-11.39 to -3.739	<0.0001
Rotenone vs. Triazine / Resveratrol	-10.52 to -2.869	0.0003
Rotenone /Triazine vs. Rotenone /Triazine / Curcumin	-8.081 to -0.4323	0.0237
Rotenone /Triazine vs. Triazine / Resveratrol	-7.211 to 0.4377	0.1030
Rotenone /Triazine / Curcumin vs. Triazine / Resveratrol	-2.954 to 4.694	0.9915

Supplementary Table 4: p-values and 95% confidence intervals (CI) for mean differences calculated for all pairwise comparisons of Antioxidant enzyme upregulation response to oxidative stress, Effects of triazine alone or in combination with curcumin or resveratrol (± rotenone exposure) at sub-lethal concentrations on CAT

Tukey's multiple comparisons test	95.00% CI of diff.	Adjusted P Value
Control vs. Triazine	-4.843 to -0.5968	0.0077
Control vs. Triazine / Curcumin	-9.117 to -4.870	<0.0001
Control vs. Triazine / Resveratrol	-7.610 to -3.363	<0.0001
Control vs. Rotenone	6.340 to 10.59	<0.0001
Control vs. Rotenone /Triazine	-1.363 to 2.883	0.9079
Control vs. Rotenone /Triazine / Curcumin	-5.813 to -1.567	0.0004
Control vs. Triazine / Resveratrol	-4.243 to 0.003209	0.0505
Triazine vs. Triazine / Curcumin	-6.397 to -2.150	<0.0001
Triazine vs. Triazine / Resveratrol	-4.890 to -0.6435	0.0066
Triazine vs. Rotenone	9.060 to 13.31	<0.0001
Triazine vs. Rotenone /Triazine	1.357 to 5.603	0.0007
Triazine vs. Rotenone /Triazine / Curcumin	-3.093 to 1.153	0.7541
Triazine vs. Triazine / Resveratrol	-1.523 to 2.723	0.9713
Triazine / Curcumin vs. Triazine / Resveratrol	-0.6165 to 3.630	0.2798
Triazine / Curcumin vs. Rotenone	13.33 to 17.58	<0.0001
Triazine / Curcumin vs. Rotenone /Triazine	5.630 to 9.877	<0.0001
Triazine / Curcumin vs. Rotenone /Triazine / Curcumin	1.180 to 5.427	0.0012
Triazine / Curcumin vs. Triazine / Resveratrol	2.750 to 6.997	<0.0001
Triazine / Resveratrol vs. Rotenone	11.83 to 16.07	<0.0001
Triazine / Resveratrol vs. Rotenone /Triazine	4.123 to 8.370	<0.0001
Triazine / Resveratrol vs. Rotenone /Triazine / Curcumin	-0.3265 to 3.920	0.1307
Triazine / Resveratrol vs. Triazine / Resveratrol	1.243 to 5.490	0.0010
Rotenone vs. Rotenone /Triazine	-9.827 to -5.580	<0.0001
Rotenone vs. Rotenone /Triazine / Curcumin	-14.28 to -10.03	<0.0001
Rotenone vs. Triazine / Resveratrol	-12.71 to -8.460	<0.0001
Rotenone /Triazine vs. Rotenone /Triazine / Curcumin	-6.573 to -2.327	<0.0001
Rotenone /Triazine vs. Triazine / Resveratrol	-5.003 to -0.7568	0.0046
Rotenone /Triazine / Curcumin vs. Triazine / Resveratrol	-0.5532 to 3.693	0.2394

Supplementary Table 5: p-values and 95% confidence intervals (CI) for mean differences calculated for all pairwise comparisons of Antioxidant enzyme upregulation response to oxidative stress, Effects of triazine alone or in combination with curcumin or resveratrol (± rotenone exposure) at sub-lethal concentrations on SOD.

Tukey's multiple comparisons test	95.00% CI of diff.	Adjusted P Value
Control vs. Triazine	-4.770 to -1.544	<0.0001
Control vs. Triazine / Curcumin	-5.826 to -2.600	<0.0001
Control vs. Triazine / Resveratrol	-5.360 to -2.134	<0.0001
Control vs. Rotenone	3.287 to 6.513	<0.0001
Control vs. Rotenone /Triazine	-1.653 to 1.573	>0.9999
Control vs. Rotenone /Triazine /Curcumin	-6.546 to -3.320	<0.0001
Control vs. Triazine / Resveratrol	-4.673 to -1.447	0.0001
Triazine vs. Triazine / Curcumin	-2.670 to 0.5563	0.3657
Triazine vs. Triazine / Resveratrol	-2.203 to 1.023	0.8985
Triazine vs. Rotenone	6.444 to 9.670	<0.0001
Triazine vs. Rotenone /Triazine	1.504 to 4.730	0.0001
Triazine vs. Rotenone /Triazine /Curcumin	-3.390 to -0.1637	0.0256
Triazine vs. Triazine / Resveratrol	-1.516 to 1.710	>0.9999
Triazine / Curcumin vs. Triazine / Resveratrol	-1.146 to 2.080	0.9675
Triazine / Curcumin vs. Rotenone	7.500 to 10.73	<0.0001
Triazine / Curcumin vs. Rotenone /Triazine	2.560 to 5.786	<0.0001
Triazine / Curcumin vs. Rotenone /Triazine /Curcumin	-2.333 to 0.8930	0.7735
Triazine / Curcumin vs. Triazine / Resveratrol	-0.4597 to 2.766	0.2722
Triazine / Resveratrol vs. Rotenone	7.034 to 10.26	<0.0001
Triazine / Resveratrol vs. Rotenone /Triazine	2.094 to 5.320	<0.0001
Triazine / Resveratrol vs. Rotenone /Triazine /Curcumin	-2.800 to 0.4263	0.2442
Triazine / Resveratrol vs. Triazine / Resveratrol	-0.9263 to 2.300	0.8100
Rotenone vs. Rotenone /Triazine	-6.553 to -3.327	<0.0001
Rotenone vs. Rotenone /Triazine /Curcumin	-11.45 to -8.220	<0.0001
Rotenone vs. Triazine / Resveratrol	-9.573 to -6.347	<0.0001
Rotenone /Triazine vs. Rotenone /Triazine /Curcumin	-6.506 to -3.280	<0.0001
Rotenone /Triazine vs. Triazine / Resveratrol	-4.633 to -1.407	0.0002
Rotenone /Triazine /Curcumin vs. Triazine /Resveratrol	0.2603 to 3.486	0.0172

Supplementary Table 6: p-values and 95% confidence intervals (CI) for mean differences calculated for all pairwise comparisons of MDA Quantification in Fly Brains, Effects of triazine alone or in combination with curcumin or resveratrol (± rotenone exposure) at sub-lethal concentrations.

Tukey's multiple comparisons test	95.00% CI of diff.	Adjusted P Value
Control vs. Triazine	-4.492 to 0.8719	0.7312
Control vs. Triazine / curcumin	-3.925 to 1.439	0.7413
Control vs. Triazine / resveratrol	-3.939 to 1.425	0.7318
Control vs. Rotenone	-29.16 to -23.80	<0.0001
Control vs. Rotenone /triazine	-21.32 to -15.95	<0.0001
Control vs. Rotenone /triazine /curcumin	-8.159 to -2.795	<0.0001
Control vs. Triazine / resveratrol	-8.999 to -3.635	<0.0001
Triazine vs. Triazine / curcumin	-2.115 to 3.249	0.9945
Triazine vs. Triazine / resveratrol	-2.129 to 3.235	0.9952
Triazine vs. Rotenone	-27.35 to -21.99	<0.0001
Triazine vs. Rotenone /triazine	-19.51 to -14.14	<0.0001
Triazine vs. Rotenone /triazine /curcumin	-6.349 to -0.9848	0.0043
Triazine vs. Triazine / resveratrol	-7.189 to -1.825	0.0005
Triazine / curcumin vs. Triazine / resveratrol	-2.695 to 2.669	>0.9999
Triazine / curcumin vs. Rotenone	-27.92 to -22.55	<0.0001
Triazine / curcumin vs. Rotenone /triazine	-20.08 to -14.71	<0.0001
Triazine / curcumin vs. Rotenone /triazine /curcumin	-6.915 to -1.551	0.0011
Triazine / curcumin vs. Triazine / resveratrol	-7.755 to -2.391	0.0001
Triazine / resveratrol vs. Rotenone	-27.91 to -22.54	<0.0001
Triazine / resveratrol vs. Rotenone /triazine	-20.06 to -14.70	<0.0001
Triazine / resveratrol vs. Rotenone /triazine /curcumin	-6.902 to -1.538	0.0011
Triazine / resveratrol vs. Triazine / resveratrol	-7.742 to -2.378	0.0001
Rotenone vs. Rotenone /triazine	5.161 to 10.53	<0.0001
Rotenone vs. Rotenone /triazine /curcumin	18.32 to 23.69	<0.0001
Rotenone vs. Triazine / resveratrol	17.48 to 22.85	<0.0001
Rotenone /triazine vs. Rotenone /triazine /curcumin	10.48 to 15.84	<0.0001
Rotenone /triazine vs. Triazine / resveratrol	9.638 to 15.00	<0.0001
Rotenone /triazine /curcumin vs. Triazine / resveratrol	-3.522 to 1.842	0.9512
